# PCR-TTGE Analysis of 16S rRNA from Rainbow Trout (*Oncorhynchus mykiss*) Gut Microbiota Reveals Host-Specific Communities of Active Bacteria

**DOI:** 10.1371/journal.pone.0031335

**Published:** 2012-02-29

**Authors:** Paola Navarrete, Fabien Magne, Cristian Araneda, Pamela Fuentes, Luis Barros, Rafael Opazo, Romilio Espejo, Jaime Romero

**Affiliations:** 1 Instituto de Nutrición y Tecnología de los Alimentos, Universidad de Chile, Santiago, Región Metropolitana, Chile; 2 Laboratoire de Biologie, Conservatoire National des Arts et Métiers, Paris, Île-de-France, France; 3 Facultad de Ciencias Agronómicas, Universidad de Chile, Santiago, Región Metropolitana, Chile; Charité-University Medicine Berlin, Germany

## Abstract

This study assessed the relative contributions of host genetics and diet in shaping the gut microbiota of rainbow trout. Full sibling fish from four unrelated families, each consisting of individuals derived from the mating of one male and one female belonging to a breeding program, were fed diets containing either vegetable proteins or vegetable oils for two months in comparison to a control diet consisting of only fish protein and fish oil. Two parallel approaches were applied on the same samples: transcriptionally active bacterial populations were examined based on RNA analysis and were compared with bacterial populations obtained from DNA analysis. Comparison of temporal temperature gradient gel electrophoresis (TTGE) profiles from DNA and RNA showed important differences, indicating that active bacterial populations were better described by RNA analysis. Results showed that some bacterial groups were significantly (*P*<0.05) associated with specific families, indicating that microbiota composition may be influenced by the host. In addition, the effect of diet on microbiota composition was dependent on the trout family.

## Introduction

The importance of intestinal bacteria in the nutrition and well being of the host has been established for several animals and was recently demonstrated in fish. It is known that the gut microbiota of fish contribute to important key functions, such as nutrition, development, immunity and xenobiotic metabolism [1], [2]. Fish harbor a microbiota that can reach 10^7^–10^11^ bacteria/g of intestinal content [3] that is dominated mainly by the phyla *Proteobacteria*, *Firmicutes* and *Actinobacteria* [2], [4]–[7]. A stable microbiota can be established after the first feeding stages, and its major components can be derived from water and egg epibiota [5], [7]. Recent reports have investigated the gut microbiota of rainbow trout using culture-independent methods [3], [6], [8]–[11]. These studies, which were conducted in Europe (Scotland, Denmark) and America (Canada, Chile), reported that the composition of the gut microbiota can be dominated by different bacterial groups. Using DGGE, Huber *et al.* [4] described *Anaerofilum*, *Carnobacterium* and *Clostridium* as the most important components of the gut microbiota. Kim *et al.* [9] found by using DGGE that uncultured *Clostridia* were the most common bands, and by cloning, >50% of the clones corresponded to *Enterobacteriaceae*. Recently, Mansfield *et al.* [10] used chaperonin (*cpn60*) instead of ribosomal RNA genes and found that >80% of the clones corresponded to *Carnobacterium*, followed by *Hafnia*, which represented approximately 10% of the clones. In another report, Navarrete *et al.* [5] used a combined approach based on 16 S rRNA gene- and *rpo*B-TTGE analysis to reveal that *Lactococcus*, *Citrobacter*, *Kluyvera*, *Obesumbacterium* and *Shewanella* dominated the intestinal microbiota. While these studies shed light on the composition of the gut microbiota, the available information does not fully clarify the factors involved in determining this composition. Exogenous and endogenous factors can affect the initial colonization and nature of the microbial composition, such as the developmental stage of the fish, the gut structure, the surrounding environment (e.g. water temperature), rearing and farming conditions [3]. The host genotype is expected to influence inter-individual variation in the intestinal microbiota. Studies in humans [12] and animals [13], [14] support the hypothesis that host-related factors are involved in the determination of the gut microbiota. The possible involvement of host genotype, particularly as it relates to immuno-phenotype, has been frequently postulated as a major influence on microbiota composition and stability, though this has been difficult to prove. In humans, it is currently unclear how the host's genetic background influences the gut microbiota because the assessment of the role of genetics in the determination of the microbiota is obscured due to environmental factors, primarily diet. The evaluation of the effect of the host on the gut microbiota can be accomplished in fish by studying different non related families of rainbow trout that are fed controlled diets.

Today, limited supplies of fish meal and fish oil for the aquaculture industry can hamper the future growth of this activity; therefore, great efforts have been made to evaluate the use of other protein and oil sources. Among the alternatives, plant-based formulations are the cheapest; many have a suitable amino acid profile and will be sustainable [15].

The aim of this study was to use molecular approaches to investigate the extent to which the rainbow trout gut microbiota is affected by the host and the inclusion of vegetable components in the diet. The overall contribution of the host to the microbiota composition was determined by analyzing full sibling individuals from four different unrelated rainbow trout families, each derived from a single pair of breeders that had been previously identified and classified in a breeding program.

## Results

### Fish performance and gut histology

Full sibling fish from four unrelated families were distributed equally in three tanks and reared under identical conditions except for their diets, which are described in Table S1. Each fish was tagged with a PIT tag that allowed individual identification and monitoring. Each group of fish was fed one of the following diets: diet D1, where 100% of the protein was provided by fish meal and 100% of the oil was provided by fish oil; diet D2, where 50% of the protein was provided by fish meal and 50% was provided by vegetable meal (corn, sunflower and soybean meal); and diet D3, where 50% of the oil was provided by fish oil and 50% was provided by rapeseed oil (Table 1). To evaluate the possible effects of the diets on the intestinal mucosa of the fish, intestines were histologically examined after two months of diet treatment according to a semi-quantitative method (Table S2). No signs of inflammation were detected in fish fed the three diets and no differences were detected between the families or diet treatments groups (Figure S1, Table S3). The villous mucosa appeared to be normal in the intestines of all the fish, with the mucosal fold forming long, finger-like structures. The enterocytes showed basal nuclei and normal round supranuclear vacuoles. Goblet cells were distributed normally among the enterocytes. The lamina propria appeared as a thin layer beneath the epithelium. The sub-epithelial mucosa, located between the basal part of the folds and the stratum compactum, showed a normal widening with no abnormal granulocyte infiltration. To assess the effects on growth, the body weight of each fish was measured after two months of diet treatment. All experimental diets were well tolerated by the fish, and no significant differences in total feed intake were observed through the end of the experiment (*P*>0.05). The inclusion of vegetable protein or oil in the diets did not significantly affect body weight gain (mean+/−SD) over the two months of the experiment (D1: 178+/−57 g; D2: 181+/−56 g; D3: 192+/−47 g; *P* = 0.889).

**Table 1:** Formulation and chemical analysis of experimental diets.

	Experimental Diets		
Ingredients (g 100 g^−1^)	Control Diet (D1)	Diet with vegetable protein (D2)	Diet with vegetable oil (D3)
Fish meal	40.0	20.0	40.0
Fish oil	23.5	24.5	11.3
Rapeseed oil	-	-	11.3
Wheat meal	16.6	14.0	16.8
Feather meal	7.0	7.0	7.0
Viscera/Entrail meal	7.3	7.3	7.3
Corn gluten	-	13.0	-
Sunflower meal	-	6.0	-
Defatted soybean meal	-	6.2	-
Vitamin and mineral premix	2.3	2.7	2.5
**Chemical composition (g 100 g^−1^)**			
Protein	41.6	43.4	43.6
Lipid	31.7	33.1	31.2
Ash	8.1	8.5	7.8
Fiber	1.3	1.3	1.3
Moisture	3.4	3.0	4.4

### Bacterial Counts

The average total bacteria from the intestinal content of fish from the different families and diet treatments are detailed in Table S4. No differences were detected among the families (*P*>0.05, Kruskal Wallis) or between fish fed different diets (*P*>0.05, Kruskal Wallis).

### Analysis of TTGE profiles derived from DNA and RNA extraction

**Figure 1:**
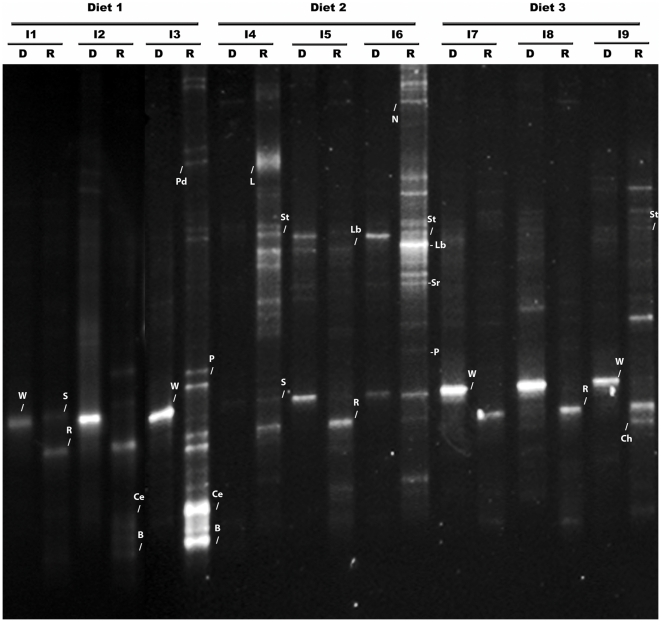
PCR-Temporal temperature gradient gel electrophoresis (TTGE) fingerprinting of the intestinal microbiota of rainbow trout (*Oncorhynchus mykiss*). Comparison of the TTGE profiles based on the amplification of the V3–V4 region of the 16 S rRNA genes from DNA extraction (D) and RNA extraction (R) from different individuals (I) from Family F1, which were fed either the control diet D1 (where 100% of the protein in the diet was provided by fish meal and 100% of the oil was provided by fish oil), diet D2 (where 50% of the protein in the diet was provided by fish meal and 50% was provided by vegetable meal (corn, sunflower and soybean meal), or diet D3 (where 50% of the oil was provided by fish oil and 50% was provided by rapeseed oil). Letters indicate some of the bacterial phylotypes described in Table 2. W: wheat component; S: *Sphingomonas*, R: *Ralstonia*, Ce: *Cellulomonas*, Pd: *Pedobacter*, B: *Blastococcus*, L: *Lactobacillus aviarus*, Lb *Lactobacillus* sp., St: *Streptococcus*, Ch: *Chelatococcus*, Sr: *Sinorhizobium*, P: *Paracoccus*, N: *Novosphingobium*.

Temporal temperature gradient gel electrophoresis (TTGE) profiles derived from a DNA analysis showed very low diversity: an intense band was observed in most of the samples (44/47). This band corresponded to the small subunit of the wheat mitochondrial 16 S rRNA gene (Figure 1 lanes D). In a few of the samples, 3 to 5 bands were observed per profile. The wheat meal was included in the three diets at approximately 16% (see Table 1). This result may explain the low diversity observed in the DNA-derived TTGE profiles, which may underestimate the diversity of the trout intestinal microbiota. To overcome this limitation, TTGE profiles were performed using RNA-extracted RT-PCR-amplified bacterial 16 S rRNA. As expected, the TTGE profiles derived from RNA showed more bands in the majority of individuals: up to 7 bands were identified (Figure 1 lanes R), and the wheat band was not observed. Thus, under our experimental conditions, analysis of the TTGE profiles derived from RNA was more informative than analysis of the TTGE profiles derived from DNA.

### Effects of host and diet on intestinal microbiota composition

**Figure 2:**
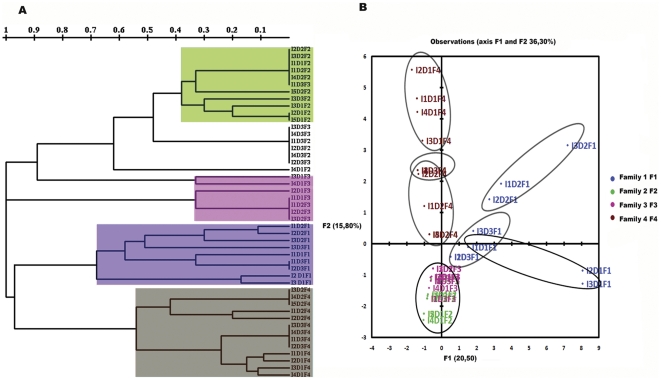
Comparison of intestinal microbiota composition between individuals (I) of the different rainbow trout (*Oncorhynchus mykiss*) families (F1, F2, F3 and F4) fed different diets (D1, D2 and D3). (A) Clustering analysis of intestinal microbiota based on distances between different groups was performed using the TREECON program with neighbor-joining method (Van de Peer and De Wachter, 1997) as described (Romero and Navarrete, 2006). (B) The principal components analysis (PCA) scores plot based on the bacterial identification data from intestinal bacterial communities.

**Figure 3:**
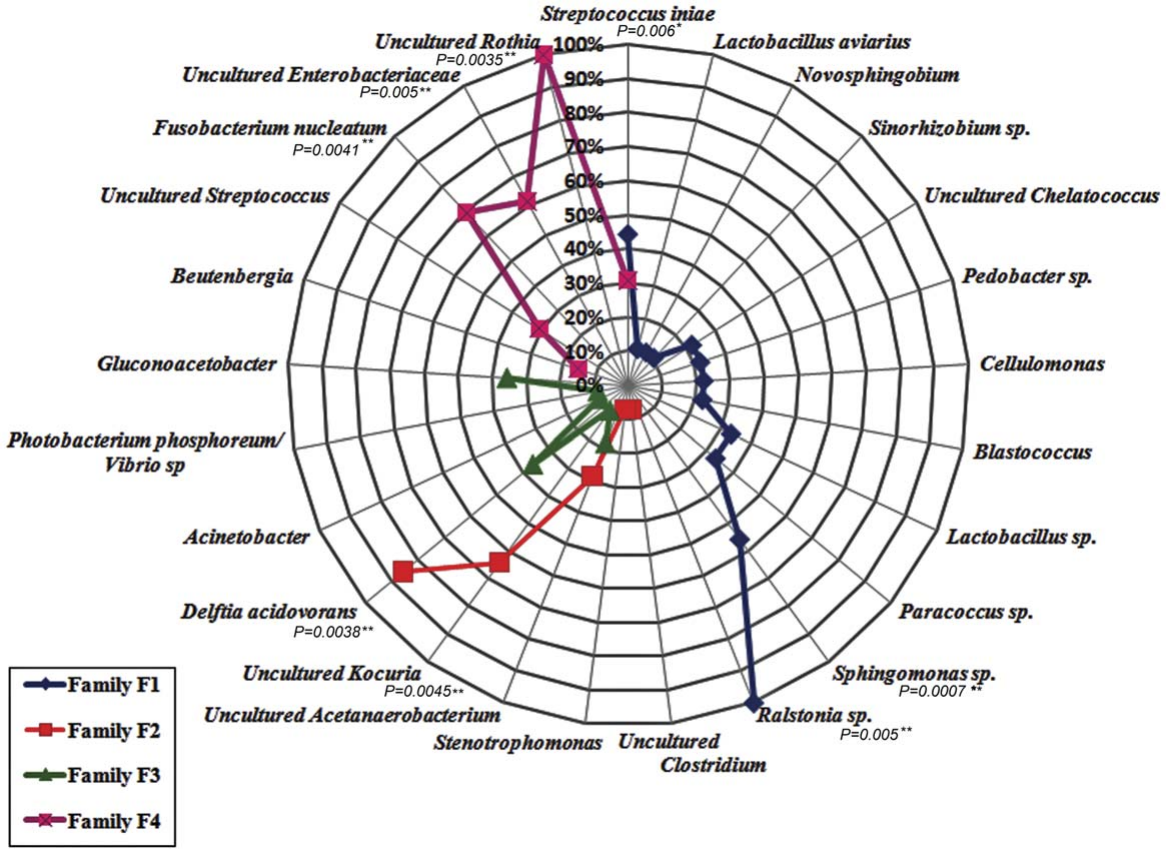
Prevalence of the different bacterial phylotypes identified in each rainbow trout family (F1, F2, F3 and 4). Bacteria identified from a specific family are represented by the same color. Prevalence values (%) show the percentage of fish in a given family that harbored a specific bacterium. The association between the bacterial phylotype and a particular family was statistical assessed.* Indicates significant association (0.005<*P*<0.05), ** Indicates highly significant association (*P*<0.005).

Dendrograms and principal components analysis (PCA) based on bacterial identification of the TTGE bands showed that the main variations in microbiota composition could be attributed to the hosts rather than the diets (Figure 2A and 2B). Analysis of PCA plots showed that host-related differences were mainly observed along the F1 axis, which accounted for 20.5% of the total variations, whereas the diets had smaller effects along F2 (15.8% of the total variations) (Figure 2B). The microbiota compositions were very different among families, although F2 and F3 were clustered together (Figure 2A and 2B) and shared some bacterial components (Figure 3). The main result was that the response of the microbiota to diet depended on the host: the four trout families responded differently to diet. Families F2 and F3 clustered together independent of diet, suggesting that these families were less affected by diet than F1 and F4 (Figure 2A and 2B). In Families F1 and F4, the variations of the microbiota were more pronounced with diet D2. This suggests that the microbiota composition from Families F1 and F4 were more susceptible to the inclusion of vegetable ingredients.

The bacterial species identified are presented in Table 2 and Figure 3. Figure 3 shows the prevalence (%) of the different bacteria in each family. A specific association was observed between certain bacterial groups and particular families. Family F1 harbored the richest microbiota: 12 bacterial species were identified (Figure 3). Among these, *Ralstonia* sp., *Sphingomonas* sp. and *Streptococcus iniae* were significantly associated with this Family (*P*<0.05). In Family F4, 6 bacterial species were identified. Among these, uncultured *Rothia*, *Fusobacterium nucleatum*, and uncultured *Enterobacteriaceae* were significantly associated with this Family (*P*<0.05). Family F3 also harbored 6 bacterial species; however, none of them were significantly associated with this Family. The microbiota of Family F2 was slightly less rich; 5 bacteria were identified. From these, *Delftia acidovorans* and uncultured *Kocuria* showed significant associations with this Family (*P*<0.05). The microbiota of Families F2 and F3 was more similar and shared some bacterial species (Table 2, Figure 3).

**Table 2:** Nearest-match identification of TTGE band sequences obtained from RNA extraction of rainbow trout intestinal microbiota with known sequences in the RDP II database.

Closest relative^a^	Phylum	Class	Fish Family (x/y)											
			F1			F2			F3			F4		
			D1	D2	D3	D1	D2	D3	D1	D2	D3	D1	D2	D3
*Sphingomonas* sp.	Proteobacteria	Alphaproteobacteria	2/3	2/3										
*Ralstonia* sp.	Proteobacteria	Betaproteobacteria	3/3	2/3	3/3									
*Paracoccus* sp.	Proteobacteria	Alphaproteobacteria	2/3	1/3										
*Pedobacter* sp.	Bacteroidetes	Sphingobacteria	2/3											
*Cellulomonas* sp.	Actinobacteria	Actinobacteria	2/3											
*Blastococcus jejuensis*	Actinobacteria	Actinobacteria	2/3											
Uncultured*Chelatococcus*	Proteobacteria	Alphaproteobacteria	1/3		1/3									
*Lactobacillus aviarius*	Firmicutes	Bacilli		1/3										
*Streptococcus iniae*	Firmicutes	Bacilli		3/3	1/3							4/4		
*Lactobacillus* sp.	Firmicutes	Bacilli		3/3										
*Novosphingobium* sp.	Proteobacteria	Alphaproteobacteria		1/3										
*Sinorhizobium* sp.	Proteobacteria	Alphaproteobacteria		1/3										
*Delftia acidovorans*	Proteobacteria	Betaproteobacteria				4/5	4/5	4/4			4/4			
Uncultured*Kocuria* sp.	Actinobacteria	Actinobacteria				4/5	5/5				1/4			
Uncultured*Clostridium*	Firmicutes	Clostridia				1/5								
*Stenotrophomonas* sp.	Proteobacteria	Gammaproteobacteria				1/5								
Uncultured*Acetanaerobacterium* *	Firmicutes	Clostridia				3/5			2/4					
*Acinetobacter* sp.	Proteobacteria	Gammaproteobacteria							1/4					
*Gluconoacetobacter* *europaeus*	Proteobacteria	Alphaproteobacteria							1/4	2/3				
*Photobacterium* *phosphoreum/Vibrio* sp.	Proteobacteria	Gammaproteobacteria							1/4					
*Fusobacterium* sp.	Fusobacteria	Fusobacteria										4/4	1/5	4/4
Uncultured *Rothia*	Actinobacteria	Actinobacteria										4/4	4/5	4/4
Uncultured*Streptococcus*	Firmicutes	Bacilli										2/4	2/5	
Uncultured*Escherichia/Shigella*	Proteobacteria	Gammaproteobacteria										4/4		4/4
*Beutenbergia* sp.*	Actinobacteria	Actinobacteria										2/4		

a :>95% identity,

* >90% identity, F: family, D: diet, x: number of individuals with this microorganism, y: total of individuals analyzed.

When vegetable meals (D2) or vegetable oil (D3) was incorporated into the diet, the richness of the bacterial composition was reduced for most families (Table 2). A higher number of bacterial species were retrieved from fish fed the control D1 (21 bacterial species), in contrast to those fed D2 and D3 (in which 14 and 9 bacterial species were identified, respectively). The incorporation of vegetable oil (D3) significantly reduced the average numbers of bacterial species per fish (*P*<0.05, Kruskal Wallis). Fish fed D1 had, on average, 3.3+/−1.8 bacterial species per fish; in contrast, fish fed D2 and D3 had 2.3+/−1.7 and 1.8+/−0.9 bacterial species, respectively.

### Effects of host and diet on dominant phyla

A total of five phyla were identified in the microbiota of intestinal trout: *Proteobacteria*, *Firmicutes*, *Actinobacteria*, *Bacteroidetes* and *Fusobacteria* (Table 2, Figure S2). Some phyla, such as *Proteobacteria*, *Firmicutes* and *Actinobacteria*, were detected in all families. Notably, those were the only phyla detected in Families F2 and F3. In addition to the three phyla mentioned, Families F1 and F4 harbored *Bacteroidetes* and *Fusobacteria* phyla, respectively. Regarding the effects of diet (Table 2), we observed that fish fed the control D1 harbored the richest microbiota, composed of all five phyla. The most evident effect of the introduction of vegetable meal (D2) or vegetable oil (D3) was the disappearance of the *Bacteroidetes* phyla in Family F1.

## Discussion

The aim of this study was to use molecular approaches to investigate the relative contributions of host background and diet to the gut microbiota composition of rainbow trout. Four unrelated trout families were studied, each consisting of individuals of similar weight, derived from the mating of one male and one female that had been previously identified and classified. Each fish was PIT-tagged, allowing different families to be reared in the same tank. Three tanks were used, each corresponding to a different diet. Diet D1 was the control diet (where 100% of the protein in the diet was provided by fish meal and 100% of the oil was provided by fish oil), and diets D2 and D3 contained either vegetable protein or vegetable oil, respectively. The microbiota composition was studied using two parallel approaches that were applied to the same samples: one approach was based on DNA analysis and the other was based on RNA analysis. The nucleic acids were obtained using separate protocols that started from the same lysates. The microbiota profiles were obtained after either PCR amplification (when DNA was the starter material) or after RT-PCR of bacterial 16 S rRNA (when RNA was the starter material).

A comparison of both approaches indicated that RNA analysis was more informative than DNA analysis because more bands were recovered in the RNA profiles. We detected the presence of a wheat component in almost all the intestinal samples analyzed using DNA. Using RNA analysis, however, it was possible to detect only bacterial RNA. These bands may correspond to ribosomal RNA from metabolically active bacteria, which may have a more important physiological role. The high number of ribosome copies, usually 1000–2000 per active bacterial cell, could explain the higher sensitivity of this approach. Therefore, the subsequent discussion will focus on the observations derived from the RNA analysis.

The small numbers of ecological studies that have described active bacterial populations in the gut are mainly focused on human microbiota and show conflicting results. Zoetendal *et al.* [16] reported that RNA and DNA profiles from the same fecal sample were very similar, in contrast to results from Sokol *et al.* [17]. As in the Sokol study, our results showed that some bands were more prominent in the RNA profiles than in the DNA profiles. Thus, some dominant bacteria described by DNA approaches could have low transcriptional activity.

The microbiota analysis from the four trout families demonstrated that the dominant bacteria belonged to the following five phyla: *Proteobacteria*, *Firmicutes*, *Actinobacteria*, *Bacteroidetes* and *Fusobacteria*. These results are consistent with earlier analyses of other freshwater and marine teleosts [7], [17]. Bacteria belonging to the *Proteobacteria* phylum, which were present at high percentages in all the families, are known to induce important responses in the host [1], [2], [14]. Members of this phylum can also exploit environmental reservoirs outside of their hosts to proliferate and maintain themselves in aquatic environments, explaining the relatively high prevalence of these bacteria [14]. Smriga *et al.* [18] have suggested that members of the *Proteobacteria*, *Bacteroidetes*, *Firmicutes* and *Fusobacteria* phyla might contribute to the digestive process by providing a variety of enzymes in fishes such as parrotfish, snapper and surgeonfish. Members of the phylum *Fusobacteria*, which are known to colonize the gut of zebrafish [19], can excrete butyrate [20] and synthesize vitamins [19] that may exert positive effects on fish health. The phylum *Actinobacteria* represents one of the largest taxonomic units among the 18 major lineages currently recognized within the domain Bacteria. Members of this phylum exhibit diverse physiological and metabolic properties, such as the production of extracellular enzymes and the formation of a wide variety of secondary metabolites [21]. Some of the members of this phylum that were recovered in this study, such as *Kocuria* sp., have been shown to positively stimulate the immune system and have recently been used as probiotics to protect against *Vibrio anguillarum* infection [22].

Studies have relied on DNA analysis as a culture-independent approach to describe the composition of the trout gut microbiota [4], [5], [9], [10]. Although each of these studies has described a different microbiota composition, a common observation is that some bacterial groups dominate the trout gut microbiota. This dominance has also been described in the gut microbiota of Atlantic salmon [23], [24]. The carnivorous diet of salmonids may explain the dominance of a low number of taxa because a recent study has indicated that diet influences the bacterial diversity of the digestive tract. Bacterial diversity increases from carnivory to omnivory to herbivory [25]. A consistent finding was recently reported in Antarctic fish, where the omnivorous *Notothenia coriiceps* exhibits greater diversity than the exclusively carnivorous *Chaenocephalus aceratus* [26]. Some of the dominant bacterial groups described in DNA studies were also detected in our RNA-based analysis. *Clostridia* described as common and abundant by Huber *et al.* [4] and Kim *et al.* [9] were observed in Families F2 and F3. In our study, lactic acid bacteria were only represented by *Lactobacilli* observed in Family F1, which contrasts with previous studies that have described the presence of abundant lactic acid bacteria represented by *Carnobacterium* [4], [10]. Until now, limited information has been published to establish the influence of the host background in determining the gut microbiota composition in fish.

The host has been claimed to play a role in shaping the gut microbiota based on observations using PCR-DGGE and cluster analyses of human [12], [16] and murine gut microbiota [13], [27], [28]. A recent study has revealed that host factors are involved in the selection of zebrafish gut microbiota [14]. This study showed that when a mouse gut microbiota is transplanted into a germ-free zebrafish, the implanted microbiota, although it resembles that of the donor animal with respect to bacterial lineages, assumes relative abundances more closely approximating the normal microbiota profile of the recipient host [14]. To date, analyses of the gut microbiota of fish, specifically salmonids, have been conducted using individuals belonging to a cohort (fish in a stock, born in the same year), and the genetic backgrounds of the fish have not been indicated. In this study, we analyzed the microbiota of four different unrelated fish families that were selected from a breeding program. Individuals from the four families were reared in the same tank and were grown under the same environmental conditions. The identification of the intestinal bacteria (Table 2, Figure 3) showed that each family harbored its own microbiota, revealing an important host influence. These results suggest that each family provides a unique habitat that selects a specific microbial community.

The host factors involved in shaping the bacterial composition are still unknown. It has recently been suggested that in a single host, differences in bacterial diversity found in the different body habitats seem to be shaped by local physiochemical properties [29]. Therefore, it has been suggested that variations in the expression of host genes involved in the establishment of these properties may affect the microbiota composition [29]. Some genetic factors associated with the immune system, such as HLA, MyD88 and IgA, and other genes related to metabolism, such as leptin, have been proposed to play a role [29].

The inclusion of some vegetable protein into fish diets may induce intestinal inflammation [30], [31] and growth disorders [32]. Inflammation could lead to alterations in gut histology and may induce changes in the microbiota composition or fish growth. We evaluated these potential effects in the different family members that were fed a diet that replaced 50% of the fish meal with vegetable proteins (diet D2). Contrary to the expected results, this diet did not affect intestinal histology (Figure S1) or fish growth, as measured after two months of treatment. This may be due to the limited proportion of soybean meal (SBM), which has been identified as a primary factor in inflammation disorders [31].

The inclusion of vegetable meal into the diet could provide fermentable carbohydrates, such as oligosaccharides of SBM, which may change the proportions of intestinal bacterial populations. Previous reports based on culture analyses have shown alterations of some bacterial groups in the gut microbiota when SBM is included in the diet. Merrifield *et al.* [33] have reported that after 16 weeks of being fed an SBM diet (50% fish meal replacement), the levels of *Psychrobacter* spp. and yeast increase considerably in contrast to *Aeromonas* spp. levels. In a study by Ringø *et al.* [34], the guts of fish that had been fed with fish meal for 12 weeks were dominated by Gram-positive bacteria of the genera *Brochothrix* and *Carnobacterium*. The Gram-negative bacteria *Chryseobacterium* spp. and *Psychrobacter glacincola* and the Gram-positive bacteria belonging to *Carnobacterium* were observed in the digestive tracts of fish fed SBM. Bakke-McKellep *et al.* [35] have described a more diverse cultivable bacterial population in Atlantic salmon that are fed SBM instead of fish meal. However, observations derived from cultivable approaches may be limited due to the low cultivability of fish gut bacteria [7], [24]. Therefore, molecular methods based on the analysis of nucleic acids were used in this study because they can provide more comprehensive data. Our results showed that the inclusion of vegetable protein in the diet reduces bacterial richness; bacteria belonging to the *Bacteroidetes* phylum were not detected in fish fed D2, and the number of bacterial species was reduced. The reason for the reduction in microbiota richness in response to the inclusion of vegetable components in the fish feed is not clear.

The literature on the effects of dietary oil types on gut microbiota composition is currently very limited. Ringø *et al.* [36] have evaluated the effects of different polyunsaturated fatty acids (FA) on cultivable lactic acid bacteria (LAB) in fresh water Arctic charr. Their results show that the frequencies of LAB are highest in the digestive tracts of fish that are fed diets with 7% linolenic acid (linseed oil) added. In contrast, lower frequencies of LAB are found in fish fed diets containing linoleic acid (coconut and soybean oil). LAB are either absent or present in low numbers in fish that are fed a control coconut oil diet, which is dominated by medium- to long-chain saturated fats. In our study, fish oil was partially replaced by rapeseed oil in diet D3. We selected this vegetable oil as the alternative oil because its ratio of linoleic acid to linolenic acid is regarded as beneficial to human and fish health, and it has an abundance of monoenoic oleic acid to maintain high growth rates [37]. Our results showed that the numbers of retrieved gut bacterial species were reduced in all the family members that were fed rapeseed oil (Table 2). The antibacterial activities of long FA against marine bacteria have recently been reviewed; the antibacterial effects depend on the structure of the FA and the type of bacteria tested [38]. The differences in the respective FA profiles and antibacterial effects of fish oil and rapeseed oil may explain the variations between the bacterial populations present in the guts of the control trout that were fed D1 (where 100% of the oil was provided by fish oil) and the trout fed D3 (where 50% of the oil was provided by fish oil and 50% was provided by rapeseed oil).

In conclusion, our results indicate that the members of a specific trout family share significant associations with certain bacterial groups, suggesting that the nature of the gut microbiota composition is influenced by the host. Moreover, the effect of diet on the bacterial microbiota of a given fish depended on the family from which that fish derived. The potential host factors, such as the genetic background, that select specific bacterial groups are currently unknown and could be the subject of future analysis. However, RNA-based analysis may be a better approach than DNA-based methods to monitor bacterial populations. It should be emphasized that this approach identifies highly transcriptionally active bacteria, which may have more important physiological roles in the trout gut than inactive bacteria.

## Materials and Methods

### Fish, diets and sampling

Full sibling fish from four unrelated families of rainbow trout (*Oncorhynchus mykiss*) were randomly selected from a genealogized breeding population PBBOT08L1F2EP obtained from the Chilean farm Hulilco Ltda (Pucón, Chile). In this breeding program, trout from a local strain, “blueback” (BB), with the highest breeding value to growth rate and percent of blue color component in the back skin were selected as breeders. The PBBOT08L1F2EP population, which was composed of 59 families, corresponds to a population with two generations of selective breeding (F2). Each families included in this study were obtained from mating one male with one female. The distribution of fish belonging to each family amongst the diet group is described in Table S1.

Rainbow trout were distributed in three outdoor tanks (volume 7000 L) from “Centro Experimental de Castro”, BioMar, Chile S.A. (42°25′18.4″S, 73°44′49.9″W; 23 meters above sea level). The fish were reared in fresh water at 8.9+/−1.5°C, with an oxygen concentration of 9.2±1.5 mg/L, and at pH 7.2±0.2. The fish were individually identified by PIT-tagging and were fed one of the following 3 diets for 2 months (Table 2): the control diet (D1): where 100% of the protein in the diet was provided by fish meal and 100% of the oil was provided by fish oil; diet 2 (D2): where 50% of the protein in the diet was provided by fish meal and 50% was provided by vegetable meal (corn, sunflower and soybean meal); and diet 3 (D3): where 50% of the oil was provided by fish oil and 50% was provided by rapeseed oil. After 2 months, all fish were killed by anaesthesia overdose. The intestinal contents from the distal intestines were kept in RNAlater. One-centimeter sections of distal intestine from each fish were taken and gently rinsed with cold (4°C) saline solution and then fixed in Bouin's solution.

### Ethics statement

This study was carried out in strict accordance with the recommendations of the Guidelines for the care and use of fish in research and the Canadian Council on Animal Care Guide to the Care and Use of Experimental Animals” (CCAC Guide, 1989). The protocol was approved by the Committee on the Ethics of Animal Experiments of INTA, University of Chile (Certificate N°: 2010-018).

### Histological analysis

A histological analysis of the distal intestine was performed to evaluate any potential inflammatory effects of the vegetable components in the diets. Intestinal samples fixed in Bouin's solution were dehydrated in accordance with standard procedures and embedded in paraffin. Transverse sections of 5-µm thickness were stained using a mixture of hematoxylin, eosin, and Alcian blue, pH 2.5. The slides were blindly evaluated using a semi-quantitative method [31], which assesses the degree of inflammation on the distal intestine using the following parameters: the morphology of the mucosal folds; the presence and size of supranuclear vacuoles; the abundance of globet cells; the infiltration of granulocytes into the lamina propria and sub-epithelial mucosa; the degree of widening of the lamina propria; and, the degree of thickening of the sub-epithelial mucosa (Table S2). All the fishes were analyzed (16 fish for D1; 16 fish for D2, and 15 fish for D3). Three transverse intestinal sections per fish were screened in entirety. The sections were photographed with a Moticam 2500 5.0 MP Live Resolution digital camera connected to a Motic BA310 light microscope. The images were processed and analyzed using Motic Images Plus 2.0 software.

### Bacterial counts, nucleic acid extractions (DNA and RNA) and PCR-TTGE

The intestinal contents stored in RNAlater were homogenized using a high-speed homogenizer (Ningbo Scientz Bio-Tech Co, China), and the homogenates were used for bacterial counts and nucleic acid extraction. For bacterial counts, the homogenates were diluted with saline solution (NaCl 0.9%), and total bacterial counts were assessed by epifluorescence microscopy using acridine orange, as previously described [39]. For nucleic acid extraction, the homogenates were suspended in RNA extraction buffer, mixed with beads and then homogenized at maximum speed in a BeadBeater (Biospec Products) for 3 min. Nucleic acids were obtained by employing separate protocols that started from the same lysate. DNA was extracted using a MoBio Powersoil Kit, and the 16 S rRNA gene was PCR-amplified and analyzed by TTGE, as described before [7]. Active bacterial populations were determined after RNA extraction, reverse transcription, PCR and TTGE analysis of the 16 S rRNA amplicons. RNA isolation was carried out using an SV Total RNA Isolation System (Promega) according to the manufacturer's instructions. First-strand cDNA synthesis was performed using an ImProm-II Reverse Transcription System (Promega) with random hexamers as primers for 1 hour at 42°C. The reverse transcriptase was then inactivated at 65°C for 15 min. PCR-TTGE was then performed as described above for DNA profiles.

### Sequencing analysis and comparison of microbiota compositions

The dominant bands were recognized in each TTGE pattern and were excised from the gel and eluted overnight in 50 µL of MilliQ water; 1 µL was used for reamplification. Amplicons were sequenced by the Macrogen USA sequencing service. The 16 S rRNA gene sequences were compared with those available in the Ribosomal Database Project II [40] (http://rdp.cme.msu.edu/seqmatch/seqmatch_intro.jsp) to ascertain their closest relatives. The sequences from TTGE bands obtained in this study are available in the GenBank database under accession numbers JN185141–JN185193. Comparison of microbiota composition was performed from bacteria identified from RNA TTGE profiles using clustering analysis, principal components analysis (PCA) and Dice's similarity coefficient (Cs) analysis [41]. Clustering analysis was performed using the TREECON program with neighbor-joining method [42] as described [7] and PCA with XLSTAT.

### Statistical analysis

Bacterial counts and numbers of bacterial species were compared using with the nonparametric Kruskal–Wallis test, with a *P*≤0.05 considered statistically significant. Statistical comparisons of the presence/absence of different bacteria across the four families were performed as follows. First, only the highest-frequency 14 bacteria present in the samples (frequencies higher than 80%). Second, to evaluate the association of specific bacteria with every family, a log-likelihood ratio test (LLR) with 3 degrees of freedom was performed [43]. Finally, a sequential Bonferroni test [44], a non-parametric technique to correct the α level when multiple non-independent tests are performed, was used. In this case, 14 k tests were performed; the first value for the “table-wide α level” was estimated as α/(k tests) = 0.05/14 = 0.00357143), the second value was computed as α/(k tests -1) = 0.00384615, the third value as α/(k tests -2) = 0.00416667, and so on, following the Rice algorithm [44].

Weight gain data in grams (ΔW = body weight day 60 – body weight day 0) from the four experimental families were analyzed by a full factorial ANDEVA using sex, diet and family as factors, followed by Student-Newman-Keuls multiple rank tests. We confirmed the normal distribution of ΔW, along with the homogeneity of variance, using Kolmogorov-Smirnov and Levene tests, respectively [45]. All statistical analyses were performed with the Statistical Package for Social Sciences (SPSS, v. 10.0).

## Supporting Information

Figure S1 **Distal intestinal epithelia of the rainbow trout (** ***Oncorhynchus mykiss*** **) in this study.** Fish were fed on the following diets: (A) control diet D1, where 100% of the protein in the diet was provided by fish meal and 100% of the oil was provided by fish oil; (B) diet D2, where 50% of the protein in the diet was provided by fish meal and 50% was provided by vegetable meal (corn, sunflower and soybean meal); and (C) diet D3, where 50% of the oil was provided by fish oil and 50% was provided by rapeseed oil. Stain: hematoxylin, eosin, and Alcian blue. Bar is 80 µm.
(DOC)

Figure S2 **Relative abundances of the different phyla identified.** Phyla identified in (A) the four unrelated trout (*Oncorhynchus mykiss*) families (F1, F2, F3 and F4) fed (B) the three diets (D1, D2 and D3).
(DOC)

Table S1 **Distribution of the rainbow trout (** ***Oncorhynchus mykiss*** **) included in this study.**
(DOC)

Table S2 **Semi-quantitative scoring system for the different parameters used to assess the degree of enteritis as described by Urán** ***et al.*** **, (2009).** The following parameters are analyzed: the changes in the morphology of the mucosal folds (MF) and supranuclear vacuoles (SNV), the abundance of goblet cells (GC), the degree of infiltration of eosinophilic granulocytes (EG), the widening of the lamina propria (LP), and the thickening of the sub-epithelial mucosa (SM).
(DOC)

Table S3 **Average value of the enteritis parameters scored using the semi-quantitative scoring system (average+/−standard error) as described in Table S2.** The following parameters were analyzed: the changes in the morphology of the mucosal folds (MF) and supranuclear vacuoles (SNV), the abundance of goblet cells (GC), the degree of infiltration of eosinophilic granulocytes (EG), the widening of the lamina propria (LP), and the thickening of the sub-epithelial mucosa (SM).
(DOC)

Table S4 **Total bacterial counts (Log_10_+/−SE) in the intestinal contents of the rainbow trout (** ***Oncorhynchus mykiss*** **), as determined by epifluorescence microscopy.**
(DOC)
