# Supplementary material for: PCR-TTGE Analysis of 16S rRNA from Rainbow Trout (Oncorhynchus mykiss) Gut Microbiota Reveals Host-Specific Communities of Active Bacteria
Source: PLoS One. 2012 Feb 29;7(2):e31335. doi: 10.1371/journal.pone.0031335 (PMC3290605; doi:10.1371/journal.pone.0031335)

**Figure S1. Distal intestinal epithelia of the rainbow trout (*Oncorhynchus mykiss*) analyzed in this study.** Fish were fed on the following diets: (A) control diet D1, where 100% of the protein in the diet was provided by fish meal and 100% of the oil was provided by fish oil; (B) diet D2, where 50% of the protein in the diet was provided by fish meal and 50% was provided by vegetable meal (corn, sunflower and soybean meal); and (C) diet D3, where 50% of the oil was provided by fish oil and 50% was provided by rapeseed oil. Stain: hematoxylin, eosin, and Alcian blue. Bar is 80 μm.

**A B C**


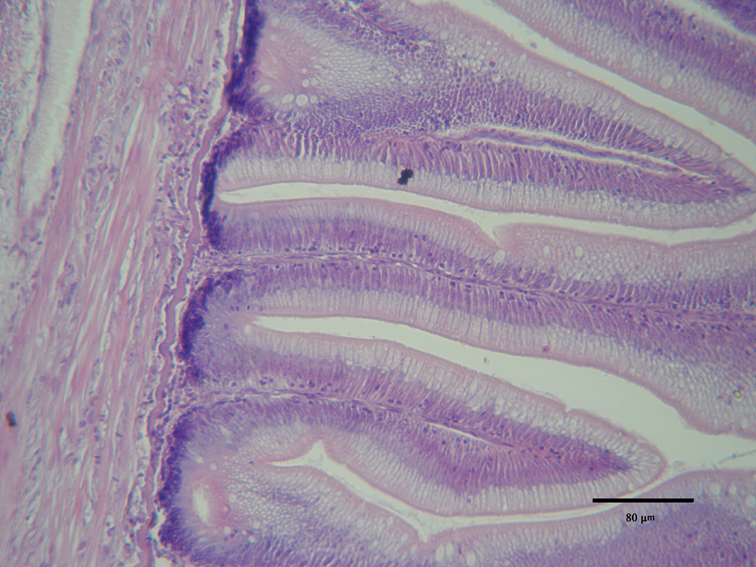

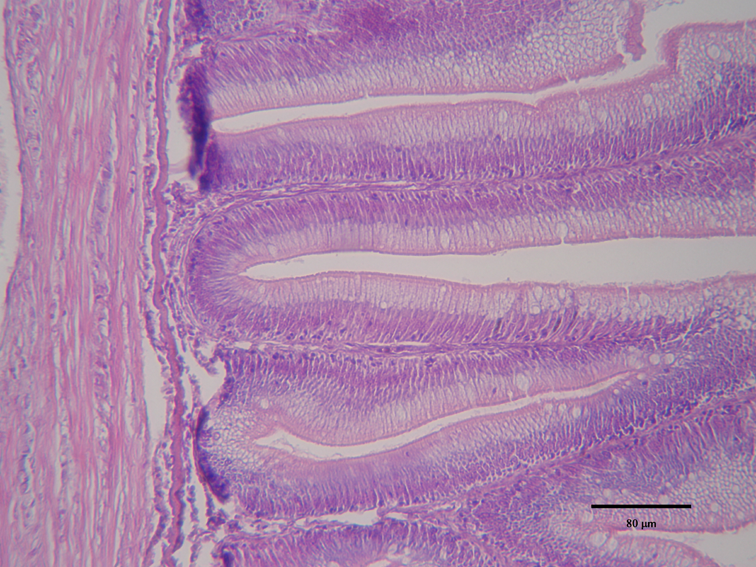

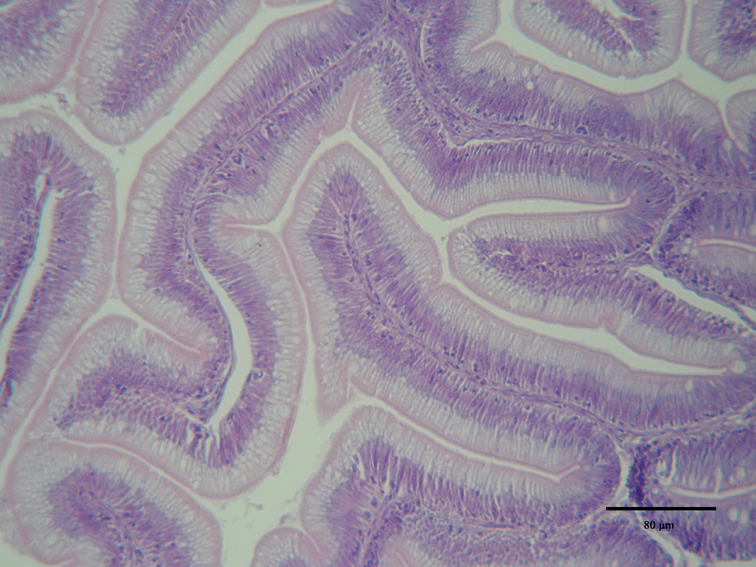

Supplement: Figure S1 — Distal intestinal epithelia of the rainbow trout ( Oncorhynchus mykiss ) in this study. Fish were fed on the following diets: (A) control diet D1, where 100% of the protein in the diet was provided by fish meal and 100% of the oil was provided by fish oil; (B) diet D2, where 50% of the protein in the diet was provided by fish meal and 50% was provided by vegetable meal (corn, sunflower and soybean meal); and (C) diet D3, where 50% of the oil was provided by fish oil and 50% was provided by rapeseed oil. Stain: hematoxylin, eosin, and Alcian blue. Bar is 80 µm. (DOC) [file pone.0031335.s001.doc]
