# Supplementary material for: PCR-TTGE Analysis of 16S rRNA from Rainbow Trout (Oncorhynchus mykiss) Gut Microbiota Reveals Host-Specific Communities of Active Bacteria
Source: PLoS One. 2012 Feb 29;7(2):e31335. doi: 10.1371/journal.pone.0031335 (PMC3290605; doi:10.1371/journal.pone.0031335)
Supplement: Figure S2 — Relative abundances of the different phyla identified. Phyla identified in (A) the four unrelated trout (Oncorhynchus mykiss) families (F1, F2, F3 and F4) fed (B) the three diets (D1, D2 and D3). (DOC) [file pone.0031335.s002.doc]

**Figure S2. Relative abundances of the different phyla identified.** Phyla identified in (A) the four unrelated trout (*Oncorhynchus mykiss*) families (F1, F2, F3 and F4) fed (B) the three diets (D1, D2 and D3).

**
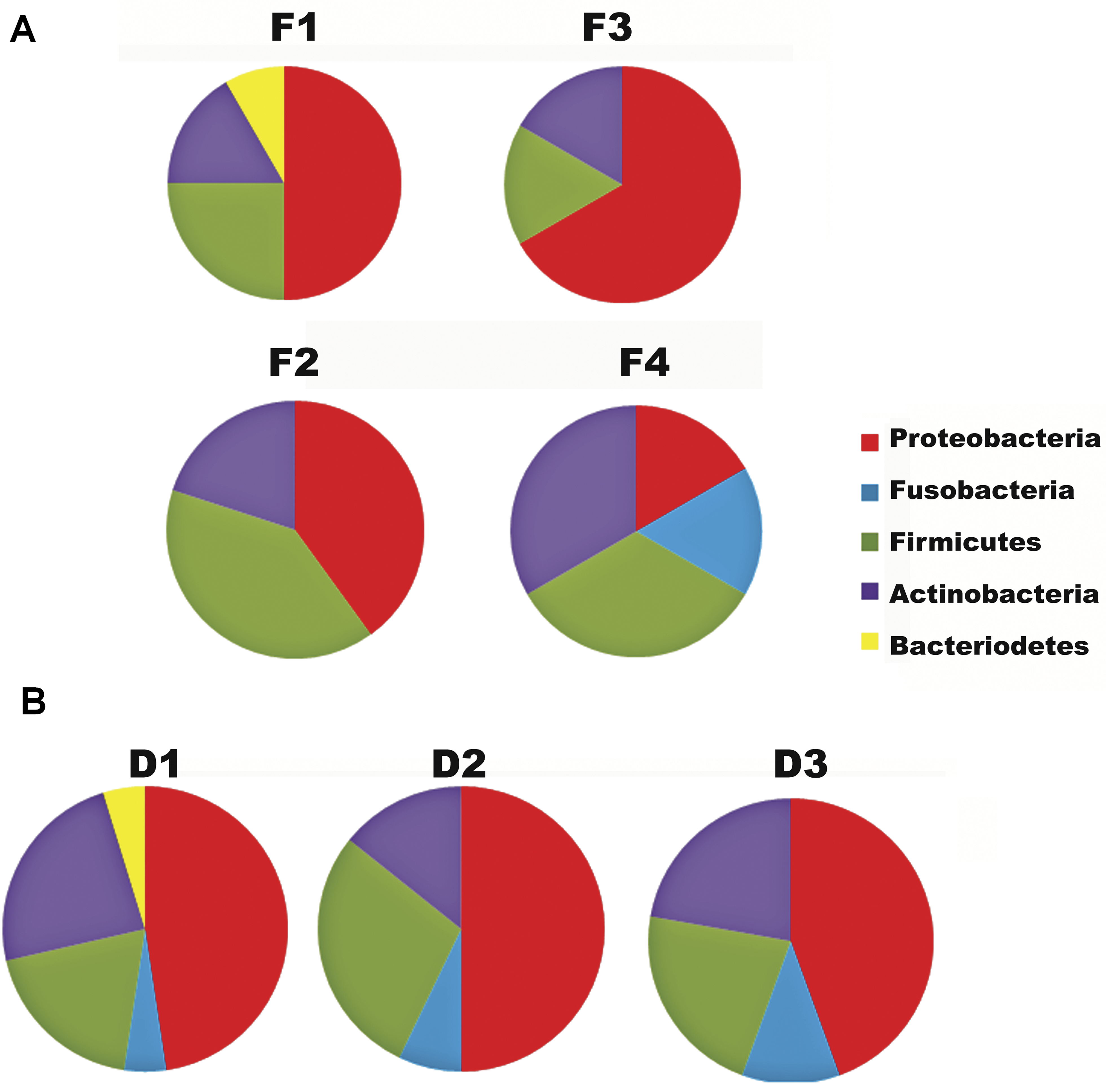
**
