# Supplementary material for: PCR-TTGE Analysis of 16S rRNA from Rainbow Trout (Oncorhynchus mykiss) Gut Microbiota Reveals Host-Specific Communities of Active Bacteria
Source: PLoS One. 2012 Feb 29;7(2):e31335. doi: 10.1371/journal.pone.0031335 (PMC3290605; doi:10.1371/journal.pone.0031335)
Supplement: Table S1 — Distribution of the rainbow trout ( Oncorhynchus mykiss ) included in this study. (DOC) [file pone.0031335.s003.doc]

**Table S1.** **Distribution of the rainbow trout (*Oncorhynchus mykiss*) included in this study**.

| **Family** * **(Numbers of fish)** | **Control Diet (D1)** | **Diet 2 (D2)** | **Diet 3 (D3)** |
| --- | --- | --- | --- |
| F1 (n = 9) | 3 | 3 | 3 |
| F2 (n = 14) | 5 | 5 | 4 |
| F3 (n = 11) | 4 | 3 | 4 |
| F4 (n = 13) | 4 | 5 | 4 |
| Total = 47 | 16 | 16 | 15 |

* Full-sib unrelated families
