# Supplementary material for: PCR-TTGE Analysis of 16S rRNA from Rainbow Trout (Oncorhynchus mykiss) Gut Microbiota Reveals Host-Specific Communities of Active Bacteria
Source: PLoS One. 2012 Feb 29;7(2):e31335. doi: 10.1371/journal.pone.0031335 (PMC3290605; doi:10.1371/journal.pone.0031335)
Supplement: Table S2 — Semi-quantitative scoring system for the different parameters used to assess the degree of enteritis as described by Urán et al. , (2009). The following parameters are analyzed: the changes in the morphology of the mucosal folds (MF) and supranuclear vacuoles (SNV), the abundance of goblet cells (GC), the degree of infiltration of eosinophilic granulocytes (EG), the widening of the lamina propria (LP), and the thickening of the sub-epithelial mucosa (SM). (DOC) [file pone.0031335.s004.doc]

**Table S2.** Semi-quantitative scoring system for the different parameters used to assess the degree of enteritis as described by Urán *et al.*, (2009). The following parameters are analyzed: the changes in the morphology of the mucosal folds (MF) and supranuclear vacuoles (SNV), the abundance of goblet cells (GC), the degree of infiltration of eosinophilic granulocytes (EG), the widening of the lamina propria (LP), and the thickening of the sub-epithelial mucosa (SM).

| **Histological analysis and Score Description** | | |
| --- | --- | --- |
| Histological structure | Score | Description |
| Mucosal folds (MF) | 1 | Basal length |
|  | 2 | Some shrinkage and bloating |
|  | 3 | Diffused shrinkage and onset |
|  | 4 | Diffused tissue disruption |
|  | 5 | Total tissue disruption |
| Supranuclear vacuoles (SNV) | 1 | Basal SNV size |
|  | 2 | Some size reduction |
|  | 3 | Diffused size reduction |
|  | 4 | Onset of extinction |
|  | 5 | No SNV |
| Goblet cells (GC) | 1 | Scattered cells |
|  | 2 | Increased number and sparsely distributed |
|  | 3 | Diffused number widely spread |
|  | 4 | Densely grouped cells |
|  | 5 | Highly abundant and tightly-packed cells |
| Eosinophilic granulocytes (EG) | 1 | Few in SM basal small quantity |
|  | 2 | Increased number in SM and some migration into LP |
|  | 3 | Increased migration into LP |
|  | 4 | Diffused number in LP and SM |
|  | 5 | Dense EG in LP and SM |
| Lamina propria (LP) | 1 | Normal size LP |
|  | 2 | Increased size of LP |
|  | 3 | Medium size LP |
|  | 4 | Large LP |
|  | 5 | Largest LP |
| Sub-epithelialmucosa (SM) | 1 | Normal SM |
|  | 2 | Increased size SM |
|  | 3 | Medium size SM |
|  | 4 | Large SM |
|  | 5 | Largest SM |
