# Supplementary material for: PCR-TTGE Analysis of 16S rRNA from Rainbow Trout (Oncorhynchus mykiss) Gut Microbiota Reveals Host-Specific Communities of Active Bacteria
Source: PLoS One. 2012 Feb 29;7(2):e31335. doi: 10.1371/journal.pone.0031335 (PMC3290605; doi:10.1371/journal.pone.0031335)
Supplement: Table S3 — Average value of the enteritis parameters scored using the semi-quantitative scoring system (average+/−standard error) as described in Table S2. The following parameters were analyzed: the changes in the morphology of the mucosal folds (MF) and supranuclear vacuoles (SNV), the abundance of goblet cells (GC), the degree of infiltration of eosinophilic granulocytes (EG), the widening of the lamina propria (LP), and the thickening of the sub-epithelial mucosa (SM). (DOC) [file pone.0031335.s005.doc]

**Table S3.** Average value of the enteritis parameters scored using the semi-quantitative scoring system (average +/- standard error) as described in Table S2. The following parameters were analyzed: the changes in the morphology of the mucosal folds (MF) and supranuclear vacuoles (SNV), the abundance of goblet cells (GC), the degree of infiltration of eosinophilic granulocytes (EG), the widening of the lamina propria (LP), and the thickening of the sub-epithelial mucosa (SM).

|  | **Family (Numbers of analyzed fish)** | | | | | | | | | | | |
| --- | --- | --- | --- | --- | --- | --- | --- | --- | --- | --- | --- | --- |
|  | F1 (n = 9) | | | F2 (n = 14) | | | F3 (n = 11) | | | F4 (n = 13) | | |
|  | **D1**  (n=3) | **D2**  (n=3) | **D3**  (n=3) | **D1**  (n=5) | **D2**  (n=5) | **D3**  (n=4) | **D1**  (n=4) | **D2**  (n=3) | **D3**  (n=4) | **D1**  (n=4) | **D2**  (n=5) | **D3**  (n=4) |
| **MF** | 1,00 +/- 0,00 | 1,00 +/- 0,00 | 1,00 +/- 0,00 | 1,00 +/- 0,00 | 1,00 +/- 0,00 | 1,00 +/- 0,00 | 1,00 +/- 0,00 | 1,00 +/- 0,00 | 1,00 +/- 0,00 | 1,00 +/- 0,00 | 1,00 +/- 0,00 | 1,00 +/- 0,00 |
| **SNV** | 1,00 +/- 0,00 | 1,00 +/- 0,00 | 1,00 +/- 0,00 | 1,00 +/- 0,00 | 1,00 +/- 0,00 | 1,00 +/- 0,00 | 1,70 +/- 0,21 | 1,33 +/- 0,33 | 1,00 +/- 0,00 | 1,07 +/- 0,07 | 1,17 +/- 0,17 | 1,00 +/- 0,00 |
| **GC** | 1,00 +/- 0,00 | 1,00 +/- 0,00 | 1,00 +/- 0,00 | 1,00 +/- 0,00 | 1,00 +/- 0,00 | 1,00 +/- 0,00 | 1,00 +/- 0,00 | 1,00 +/- 0,00 | 1,00 +/- 0,00 | 1,00 +/- 0,00 | 1,00 +/- 0,00 | 1,00 +/- 0,00 |
| **EG** | 1,00 +/- 0,00 | 1,00 +/- 0,00 | 1,00 +/- 0,00 | 1,00 +/- 0,00 | 1,00 +/- 0,00 | 1,00 +/- 0,00 | 1,00 +/- 0,00 | 1,00 +/- 0,00 | 1,00 +/- 0,00 | 1,00 +/- 0,00 | 1,00 +/- 0,00 | 1,00 +/- 0,00 |
| **LP** | 1,00 +/- 0,00 | 1,00 +/- 0,00 | 1,00 +/- 0,00 | 1,00 +/- 0,00 | 1,00 +/- 0,00 | 1,00 +/- 0,00 | 1,00 +/- 0,00 | 1,00 +/- 0,00 | 1,00 +/- 0,00 | 1,00 +/- 0,00 | 1,00 +/- 0,00 | 1,00 +/- 0,00 |
| **SEM** | 1,60 +/- 0,24 | 1,17 +/- 0,17 | 1,00 +/- 0,00 | 1,67 +/- 0,23 | 1,00 +/- 0,00 | 1,00 +/- 0,00 | 1,00 +/- 0,00 | 1,00 +/- 0,00 | 1,00 +/- 0,00 | 1,20 +/- 0,11 | 1,00 +/- 0,00 | 1,00 +/- 0,00 |
