# Supplementary material for: PCR-TTGE Analysis of 16S rRNA from Rainbow Trout (Oncorhynchus mykiss) Gut Microbiota Reveals Host-Specific Communities of Active Bacteria
Source: PLoS One. 2012 Feb 29;7(2):e31335. doi: 10.1371/journal.pone.0031335 (PMC3290605; doi:10.1371/journal.pone.0031335)
Supplement: Table S4 — Total bacterial counts (Log10+/−SE) in the intestinal contents of the rainbow trout ( Oncorhynchus mykiss ), as determined by epifluorescence microscopy. (DOC) [file pone.0031335.s006.doc]

**Table S4.** Total bacterial counts (Log_10_ +/- SE) in the intestinal contents of the rainbow trout (*Oncorhynchus mykiss*), as determined by epifluorescence microscopy.

| **Family (Numbers of analyzed fish)** | **Control Diet (D1)** | **Diet 2 (D2)** | **Diet 3 (D3)** |
| --- | --- | --- | --- |
| F1 (n = 9) | 7,92 +/- 0,10 | 7,80 +/- 0,21 | 7,91 +/- 0,16 |
| F2 (n = 14) | 8,12 +/- 0,00 | 7,63 +/- 0,17 | 7,82 +/- 0,15 |
| F3 (n = 11) | 7,70 +/- 0,08 | 7,95 +/- 0,10 | 7,80 +/- 0,03 |
| F4 (n = 13) | 7,76 +/- 0,11 | 8,02 +/- 0,06 | 7,83 +/- 0,12 |
